# The Influencing Factors of Art Graduates’ Entrepreneurship by Logistic Regression Analysis From the Perspective of Entrepreneurial Mentality

**DOI:** 10.3389/fpsyg.2022.870448

**Published:** 2022-06-22

**Authors:** Yanmin Li, Xin Wang, Huizhen Long, Lele Ye, Yifang Gao

**Affiliations:** ^1^Pan Tianshou College of Architecture, Art and Design, Ningbo University, Ningbo, China; ^2^College of Journalism and Communication, Shih Hsin University, Taipei City, Taiwan; ^3^School of Hotel and Tourism Management, The Hong Kong Polytechnic University, Hong Kong, Hong Kong SAR, China; ^4^Zhijiang College of Zhejiang University of Technology, Shaoxing, China; ^5^College of Art and Design, Shanghai Jian Qiao University, Shanghai, China

**Keywords:** psychology, logistic regression analysis, art design, entrepreneurial team, college student’s entrepreneur

## Abstract

The purpose of this study was to understand the influence mechanism of college students’ entrepreneurial intention in view of the increasing number of college students at present to alleviate college students’ employment competition. The psychological factors that influence the entrepreneurial tendency of art graduates were analyzed and studied. First, venture capital and factors affecting entrepreneurial performance were analyzed. Second, the coefficient calculation is carried out for college students majoring in art through the regression analysis of the logistic model. Finally, a team entrepreneurial performance questionnaire was designed, and team reward levels were discussed. The results show that the logistic model can well reflect the real situation of the respondents. The significance level of the entrepreneurial team was 0.02, which was correlated. Additionally, corresponding suggestions were put forward according to the questionnaire results. Clear team goals, assignment of tasks to members, good pressure resistance, and psychological quality of members are necessary qualities for successful entrepreneurship. This conclusion provides a certain theoretical basis for the current college students’ entrepreneurial learning and a reliable inspiration for helping college students to successfully start a business.

## Introduction

The problem of art students’ employment difficulties has become increasingly prominent due to university enrollment expansion, design and art students’ mentality, industry nature, employment form, and other factors. Therefore, to solve the problem of employment of design art graduates, it is necessary to actively explore employment guidance, talent training, and service system that is compatible with the employment characteristics of design art students. The expansion of higher education schools in China has gradually increased the number of college students. Many college graduates apply for jobs every year. However, with the continuous development and improvement of various industries, China has experienced overcapacity. Job supply is far less than the demand for graduates. Therefore, the employment difficulty in China has emerged (Liu et al., 2021). Due to the lack of social experience of college students, the entrepreneurial success rate of college students generally remains at a low level (Wathanakom et al., 2020). Among them, the number of art-oriented occupations is small, and the degree of association between art majors and other majors is not high, so the employment of art students is very difficult. Art students pursue personality display, and the traditional occupation is not accepted by art students. Therefore, art students’ entrepreneurship has become the preferred way of employment for art graduates. Entrepreneurship education with culture and art management as the main body should be carried out with a certain unit as the main body to make it flourish in a school, instead of each department being busy and using its own limited resources. Under unified management, education combines innovation and entrepreneurship in their respective artistic fields and develops a creative culture. Taking the Dalian Art Institute as an example, the main unit can be the School of Culture and Art Management. Talents in culture and art management can popularize management knowledge for art students and help them run entrepreneurial projects. E-commerce majors can combine the “Internet + “ upsurge to do publicity and promotion jobs on the internet. A diversified and excellent entrepreneurial team has been formed to streamline operations and management to maximize resource utilization. Therefore, with the College of Culture and Art Management as the main body, the mode and countermeasures of college students’ innovation and entrepreneurship education should be considered.

However, the entrepreneurial environment of art graduates has not improved because of the small number of social occupations. Jones et al. (2019) pointed out the paradox caused by the multiple goals of remote art centers and social enterprises. These goals are often conflicting. How the social, commercial, and cultural logic of art centers leads to the persistent dilemma has become an obstacle to the development of art innovation (Jones et al., 2019). For this dilemma, art students cannot be abandoned because art entrepreneurship has less effect on society. Instead, art entrepreneurship should be strongly supported. For example, to understand the current use of social media in entrepreneurship courses, Wu and Song explore the use and satisfaction of social media in entrepreneurship courses from the perspective of learners. This result reveals that trust, profit, learning, and social interaction are three factors that satisfy learning mentality. In particular, the element of trust deserves further discussion in curriculum research (Wu and Song, 2019). Increasing entrepreneurs’ confidence is an important factor in entrepreneurial success. Radoslaw et al. (2018) thought that the high score of narcissism and competition indicates the vulnerability of self-esteem, while the high score of admiration indicates the best self-esteem. Competition is between fragile narcissism and admiration. This supports its positioning in the self-importance dimension of the narcissistic model (Radoslaw et al., 2018). Wu et al. (2019) believed that narcissism or spiritual quality has a certain impact on educational entrepreneurship and practice. Marshall et al. (2020) used an experimental and small-illustrator-based method to investigate entrepreneurs and obtained multiple related indicators such as resources and higher levels of happiness, life satisfaction, and psychological wellbeing. Some studies have established a structural model to form students’ entrepreneurial intentions. The survey results found that entrepreneurial attitudes, social capital, and psychological capital synergistically affect the entrepreneurial willingness of science and engineering students. Psychological capital has a positive partial mediation effect on the relationship between entrepreneurial attitude orientation and entrepreneurial intention (Mahfud et al., 2020). To sum up, most of the current studies on such issues are qualitative analysis, and some only focus on analyzing one or two factors, without comprehensive consideration of various factors. Some questionnaires are issued in small numbers and lack credibility. Thus, the psychological level of entrepreneurs has a significant impact on the success of entrepreneurship. At present, most studies focus on the entrepreneurial intention of graduates, and there are few studies on the psychological factors of graduates themselves. Therefore, this study studies graduates’ entrepreneurial intentions from the perspective of mentality.

To create a favorable entrepreneurial or employment environment for art graduates, the factors contributing to this difficult entrepreneurial situation should be discussed, and strategies to improve the environment should be analyzed. To sum up, in the era of mass entrepreneurship and innovation, it is of great significance for social and economic development to analyze the entrepreneurial willingness of college students to understand its impact mechanism. Therefore, the innovation lies in the analysis of the psychological level of artistic entrepreneurs from the perspective of mentality. First, the related concepts of entrepreneurship and mentality are expounded. After that, a questionnaire is designed and the entrepreneurial team of art majors is investigated. The related psychological and entrepreneurial problems of entrepreneurs are analyzed under the premise of ensuring the validity of the questionnaire. Subsequently, statistical analysis is used to analyze the survey results. Multivariate logistic regression analysis is used to explore the influence of various factors on art entrepreneurs and investigate data. Through several years of exploration, the cultivation of innovative and entrepreneurial talents in art colleges and universities has achieved certain results. There are still a small number of innovative talents, and it is difficult to meet the needs of national economic development and social development. Art colleges and universities should seize new opportunities, meet new challenges, cultivate more innovative and entrepreneurial talents in art, and use talents to drive industrial development. Additionally, the prosperity of the cultural industry has further prompted colleges and universities, especially art colleges, to adapt to their development and reform their own talent training methods and achieve common development.

## Materials and Methods

### The Theoretical Basis of Social Venture Capital and Entrepreneurship

At the end of the last century, “social capital” was first proposed (Redondo and Camarero, 2019). This attracted wide attention in the academia at that time. Many experts analyze the nature of social capital from the aspects of social competition, management, and connotation, which promotes the further improvement and development of this concept.

The design field of this concept is very extensive. First, social entrepreneurial capital mainly designs individual social networks and resources. In brief, it can be divided into four factors, namely, relationship, resources, functional characteristics, and ability. The quantitative method is widely used in the study of social venture capital. Among them, it includes the naming method and positioning method (Miković et al., 2020). The naming method is to make the respondents think about which kind of person to seek solutions when they encounter problems. The structure of the respondents selected in this way is called discussion network. Ways to solve problems are investigated by assuming integration scenarios (such as job search and medical care). The social entrepreneurship network is constructed according to the occupation, economic ability, education level, age, and relationship of the respondents.

**FIGURE 1:**
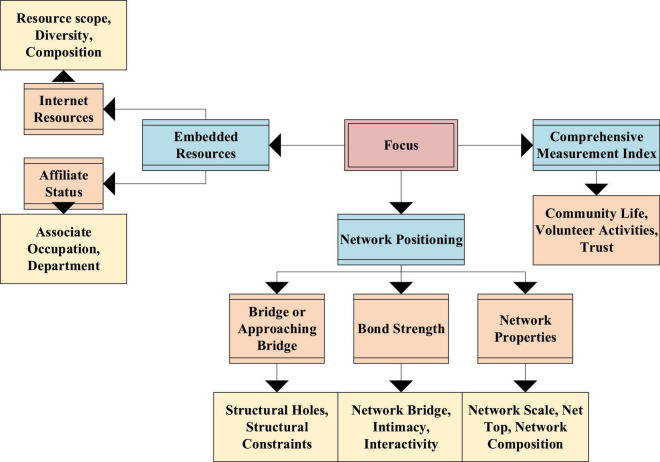
Social entrepreneurship capital test indicators.

Since the nomenclature law cannot be applied in certain specific social capital networks, it cannot reflect the total amount of entrepreneurial capital. Therefore, scholars put forward the positioning method on this basis. By testing the distribution of individual occupations in a specific social network, the characteristics of individual occupation network are established, and resource information is obtained. At present, the quantitative research of social entrepreneurial capital refers to the test indicators in Figure 1 using the naming method and positioning method.

In Figure 1, the social entrepreneurial capital test indicators mainly include three aspects, namely, embedded resources, network positioning, and comprehensive measurement indicators. Among them, embedded resources include network data and associate status measurement methods. Network positioning includes three measurement methods, namely, bridge or near bridge, bond strength, and network properties. The comprehensive measurement indicators mainly test social entrepreneurial capital through participation in community life, public affairs, volunteer activities, and mutual trust.

### Factors Influencing Entrepreneurial Tendency

**FIGURE 2:**
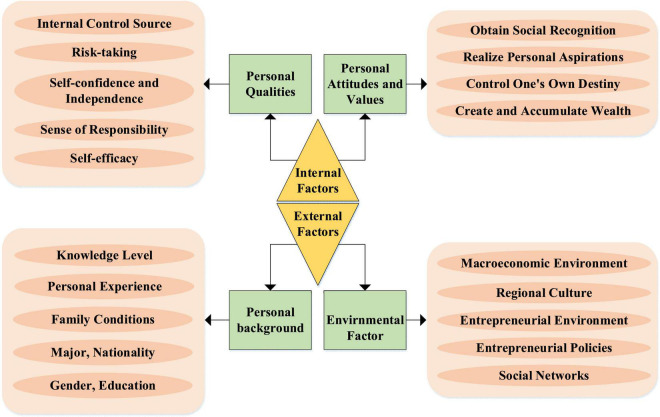
Factors affecting entrepreneurial propensity.

From the current literature analysis, there are three views about the influencing factors of entrepreneurial tendency, namely, (1) psychological perspective; (2) economic perspective; and (3) education perspective. In this research, the questionnaire is the research method adopted by most studies. The applicability of the questionnaire method can be positively surveyed. The entrepreneurial propensity model is divided into two dimensions, namely, entrepreneurial intention and entrepreneurial feasibility (Reissová et al., 2020). In this model, entrepreneurial intention is the entrepreneurial interest in creating a new career. Feasibility is the possibility that individuals think they can successfully create a career within their ability. The specific factors are shown in Figure 2. The influencing factors of entrepreneurial tendency are divided into internal factors and external factors. Among them, internal factors include three categories, namely, personal traits, personal attitudes, and values. External factors include personal background and environmental factors.

The subjects involved in entrepreneurial propensity include pedagogy, sociology, and psychology (Ndofirepi, 2020). The purpose of pedagogy is to research and offer courses to improve entrepreneurial propensity. The purpose of sociology is to study the influence of social culture and environment on entrepreneurial propensity. Mentality focuses on the impact of personal attitudes toward entrepreneurial propensity. Sociology and pedagogy have different degrees of influence on mentality.

**FIGURE 3:**
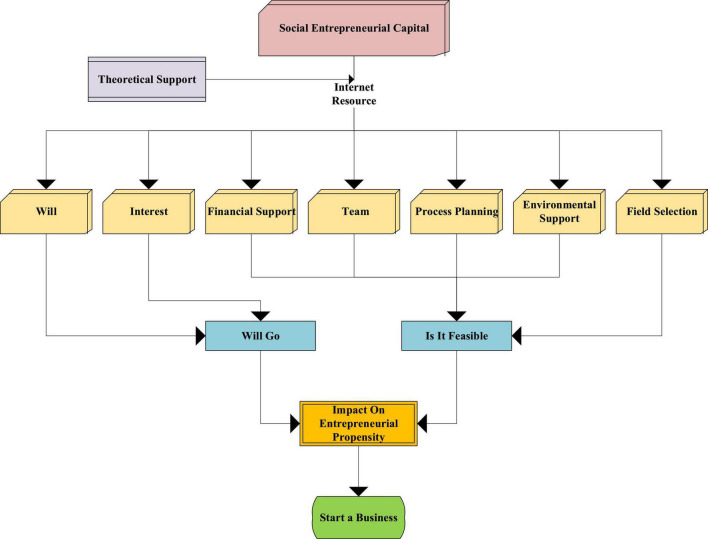
The influence process of entrepreneurial factors.

There are seven factors that affect entrepreneurial propensity, namely, entrepreneurs’ willingness, interest, financial support, team support, emotional support, field selection, and process planning (Bigos and Michalik, 2020). These seven factors are used as the basis of the model. The influence process of these factors on entrepreneurs is shown in Figure 3.

In Figure 3, the factors influencing entrepreneurship are analyzed, and they are divided into two dimensions, namely, entrepreneurial willingness and entrepreneurial feasibility. Among them, entrepreneurial intention refers to the desire and interest of entrepreneurs to create a new enterprise. Entrepreneurial feasibility refers to the feasibility of an entrepreneur to create a new enterprise within one’s own capabilities, a factor related to financial support and the adequacy of potential partners, and a kind of entrepreneurial readiness. Based on these classifications, case interviews are used to identify factors of entrepreneurial propensity and to discover the relationship between social capital and entrepreneurial propensity and to explore the relationship between various factors of entrepreneurial propensity.

**FIGURE 4:**
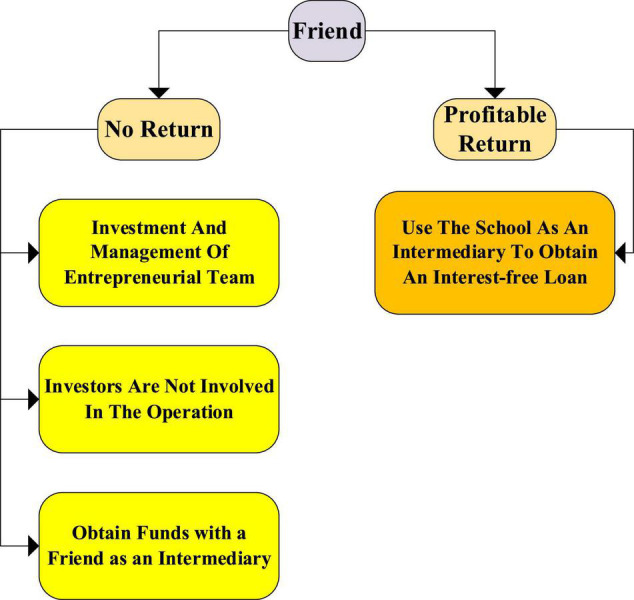
Sources of funds for entrepreneurs.

Among the above factors, funding is the foundation of entrepreneurship and the starting point of entrepreneurship. However, for graduating college students, funding is the first difficulty in starting a business (Anjum et al., 2021). Figure 4 is the source of financial support for entrepreneurs. Entrepreneurship requires funds to produce products, funds for advertising, payment of employee salaries, and training of employees. Relying solely on personal ability, the funding problem is difficult to overcome.

A good team can reduce the difficulty of starting a business. The team is the complement of personal skills and promotes personal strengths. The group needs to be led by someone recognized by everyone. The entrepreneurial team needs to have a common entrepreneurial goal for internal collaboration. A good team plays a decisive role in an enterprise. In the major well-known industries, 90% of the companies are organized by teams. The composition of the entrepreneurial team also requires the accumulation of personal connections. The resources owned by a mature team will become the driving force for the operation of the enterprise (Twum et al., 2021).

Chinese people generally believe that stable work is a better choice than self-employment. Entrepreneurship is a process full of randomness. For entrepreneurs, family support has a significant impact on entrepreneurs. Emotional factors can affect entrepreneurs’ funds in some cases. The support of affluent families for entrepreneurship far exceeds that of ordinary families because wealthy families can bear the consequences of entrepreneurial failure better than ordinary families (Gujrati et al., 2019). Overall, family support has a greater impact on entrepreneurs.

Field selection for entrepreneurship. Generally, entrepreneurs will choose industries related to their majors to start their businesses. Among them, there are also entrepreneurial ventures in other industries out of interest. The success of this kind of entrepreneurship depends on one’s own humanistic, financial, and professional knowledge. In addition, there are also entrepreneurs who choose to start a business because of their richer network resources in a certain industry (Rampasso et al., 2021).

Location, information, material sources, and experience are the basic requirements for successful entrepreneurship. On this basis, rational use of resources in hand can effectively increase the success rate of entrepreneurship and reduce the risk of entrepreneurial failure.

**FIGURE 5:**
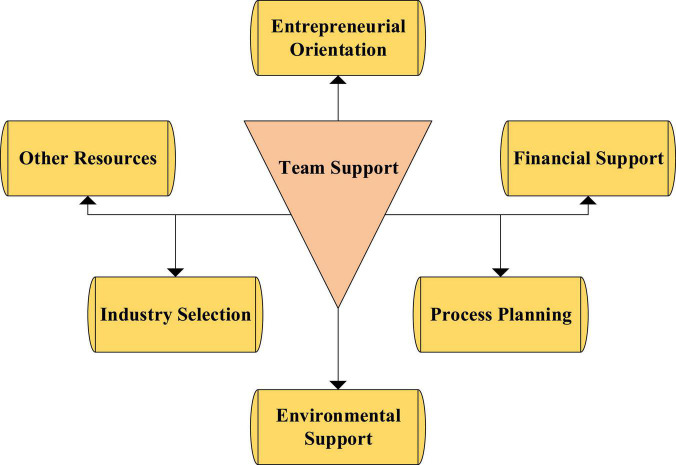
Structure diagram of entrepreneurial propensity.

These factors take the entrepreneurial team as the main influence, presenting a radial structure radiating from the center to the outside, as shown in Figure 5. The reasons for the emergence of the entrepreneurial team mainly include factors such as entrepreneurial orientation, financial support, process planning, environmental support, industry selection, and other resources.

### Application Analysis of Logistic Regression Analysis Model in Entrepreneurial Intention Questionnaire

Many phenomena can be divided into two possibilities or can be attributed to two states. These two states are represented by 0 and 1, respectively. If a phenomenon represented by 0–1 is explained by causality, it may be applied to logistic regression.

Logistic regression is divided into binary logistic regression and multivalue logistic regression. First, an example is used to describe binary logistic regression. Then, multivalue logistic regression is further illustrated. The main operation steps of logistic regression analysis are as follows: (1) first, the regression object and test standard need to be determined; (2) the independent variable and dependent variable are selected for system initialization; (3) the independent variable is divided into two categories. When necessary, dummy variables need to be set, and dummy variables are used as categorical covariates; (4) irrelevant data and impurities are removed; and (5) regression analysis results are obtained. Whether the sign is less than 0.05, it is judged whether the independent variable influences the dependent variable, and the regression equation can be written according to the value of each variable in step (3).

Before the regression model is used, the number of respondents needs to be determined, that is, to select the appropriate number of samples. Too many samples will waste excess resources, while too few samples will cause the consequences of inaccurate survey results. Therefore, the equation for selecting a suitable sample size is as follows:


(1) $$n=\frac{1}{\left(\frac{1}{N}+\frac{d^{2}}{\mu_{\frac{\alpha }{2}}^{2}\times s^{2}}\right)}$$

After equation (1) is simplified, equation (2) is obtained as follows:


(2) $$n=\frac{N\,\mu_{\frac{\alpha }{2}}^{2}\times s^{2}}{N\,d^{2}+\mu_{\frac{\alpha }{2}}^{2}\times s^{2}}$$

Among them, N is the overall scale of the survey object, that is, the total number of survey objects that can be selected as survey objects in the survey site. d is the limit sampling error when conditions allow (Braun et al., 2019). It is a constant determined according to the on-site situation. *s*^2^ = px(1–p), p is the proportion of the sample, generally valued 0.5. After that, the investigated model is evaluated, and the prediction result is obtained. After the predicted value and the true value are compared, the generalization ability of the model is judged. Logistic regression is mainly divided into three categories. The first is logistic regression, where the dependent variable is binary. This regression is called binomial logistic regression. The second is the logistic regression with unordered multiclassification of the dependent variable, such as which product to choose. This regression is called multinomial logistic regression. The third is logistic regression, where the dependent variable is an ordinal multiclassification. In general, the results of the comparison between logistic predicted results and the real results are shown in Table 1.

**TABLE 1:** Logistic model prediction results.

Real result forecast result	0	1
0	TN	FN
1	FP	TP

Through Table 1, four quantifications are defined: TN is the number of samples whose actual results are inversely proportional to the predicted results; TP is the number of samples whose actual results are positively proportional to the predicted results; FP is the actual result and it is a negative example, but the predicted result is a positive example; FN is the sample size where the actual result is a positive example, but the predicted result is a negative example. These four quantifications can be used to express the accuracy of the model (Feng et al., 2019), as shown in the following equation:


(3) $$A\,C\,C=\frac{T\,P+T\,N}{T\,P+T\,N+F\,P+F\,N}$$

The accuracy of the model is higher than 60%, indicating that the accuracy of the model is good. However, in some experiments, due to too many influencing factors, the above formulas cannot directly explain the accuracy of the model. Therefore, other quantities are introduced into auxiliary judgment. F1 is the weighted harmonic average of accuracy and recall. P represents the accuracy of the model, which is the ratio of the actual results to the predicted results in the model, as shown in the following equation:


(4) $$P=\frac{T\,P}{T\,P+F\,P}$$

R represents the recall rate. The actual result of R is a positive example. After model prediction, R is also the ratio of positive cases, as shown in the following equation:


(5) $$R=\frac{T\,P}{T\,P+F\,N}$$

The definition of F1 is shown in the following equation:


(6) $$F_{1}=\frac{2\,P\,R}{P+R}$$

For the unbalanced data in the experiment, it needs to be processed by over sampling or under sampling. Over sampling is to reorganize the data arrangement rule to solve the unbalanced data and improve the classification performance by increasing the number of samples. Over sampling has the problem of overfitting. The reason is that additional data that have not been accurately tested is added. Therefore, the synthetic minority over sampling technique (smote) algorithm is introduced (Shen et al., 2021). The basic theory of this algorithm is to synthesize a few new samples and draw samples from adjacent random samples. A point on the line between the two samples is extracted as a new minority sample. The essence of under sampling is to extract some samples from many data samples as representatives. However, this method will cause some important data information to be lost, and the data information cannot be fully utilized. Therefore, cluster-based down sampling is used to solve this problem. Logistic regression, like multiple linear regression, needs to analyze whether the data can use a logistic regression model before application. Instead of the dependent variable being a categorical variable, logistic regression can be used directly. Some conditions still need to be considered. The first condition should be to see what kind of relationship is between the independent variable and the dependent variable. In multiple linear regression, the independent variable is required to have a linear relationship with the dependent variable.

### Questionnaire Design and Survey Analysis Based on Entrepreneurial Intention

**FIGURE 6:**
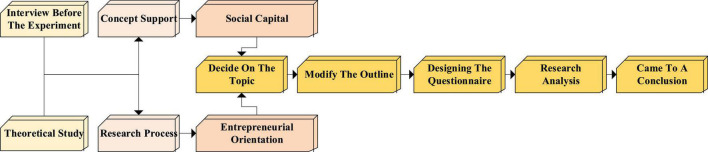
Technical roadmap of the questionnaire.

First, relevant theoretical concepts in the literature are searched for and learned. Second, relevant experts are preinterviewed (Valdez et al., 2021). The tendency of entrepreneurship is regarded as the main research direction. The intervention of social entrepreneurial capital is used as an auxiliary item, as shown in Figure 6. Some art entrepreneurs are surveyed. The influencing factors in the entrepreneurial process are open for discussion, and the practice of entrepreneurial propensity and social entrepreneurial capital is explored. The outline is revised and improved. According to the interview results, the questionnaire is designed, distributed, collected, and counted.

In Figure 6, the interview outline is designed during the questionnaire design. Based on the literature review and according to the research objectives, the respondents talk openly about various aspects of their entrepreneurial tendencies. Based on the preinterview, there is a certain relationship between entrepreneurial tendency and social capital. The questionnaire is adjusted and determined.

According to the influencing mechanism of the performance of the entrepreneurial team of college students majoring in art, the questionnaire is designed mainly for the influencing factors of the entrepreneurial team. For the intervention of entrepreneurial propensity, the Appendix is a designed questionnaire. The questionnaire mainly includes three aspects, namely, the management of the entrepreneurial team (questions 1–10), the effectiveness of the members’ entrepreneurship (questions 11–19), and the leadership of the person in charge (questions 20–30).

The questionnaire is tested for validity. The SPSS 25.0 statistical analysis software was used (Han and Ellis, 2020) for testing. The test results are shown in Table 2.

**TABLE 2:** Questionnaire validity test.

Inspection aspect	Is the investigation objective clear	Whether the team resources are sufficient	Is task feedback real-time
Coefficient of validity	0.869	0.902	0.612

The test validity coefficients of these three aspects are all greater than 0.6, indicating that the overall validity of the questionnaire is good.

Senior-year students of art majors from Xi’an Conservatory of Music are selected as samples. In total, 50 entrepreneurial teams are selected, and 370 questionnaires are distributed. Five days later, 340 questionnaires are recovered. The final valid questionnaire is 290.

## Results and Discussion

### Coefficient Value Test of Logistic Regression Model

**FIGURE 7:**
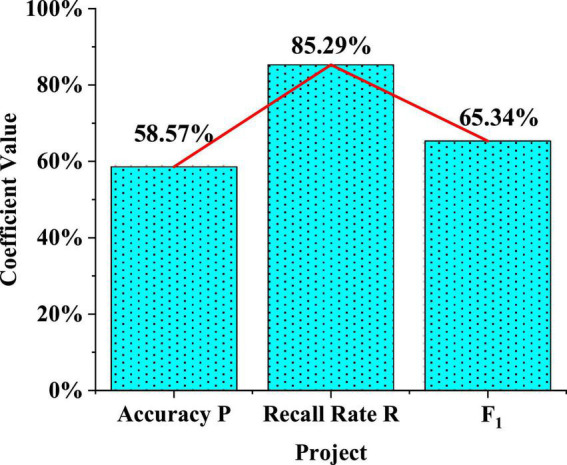
Logistic model performance test.

The precision rate calculation equation of the logistic model is used to calculate and test the performance coefficient of the logistic regression model of the questionnaire, as shown in Figure 7.

In Figure 7, the accuracy P of the logistic model is 58.57%. Although the accuracy rate is not high, the test is performed within the acceptable error range. The recall rate R is 85.29%. Under normal circumstances, the precision rate and the recall rate are mutually restricted. The accuracy rate is relatively low, the recall rate will increase, and there is a restrictive relationship between the two. The value of F1 is 65.34%, which is higher than 60%, indicating that the model has strong generalization ability. This logistic model can well show the real situation of the survey object. Parameter estimates for each variable include estimates of intercepts, as well as standardized parameter estimates. The standardized estimated value is generally used to measure the influence of different independent variables on the dependent variable (limited to continuous independent variables, meaningless for standardized parameter estimation of categorical independent variables). Statistics are used to check the significance of non-zero parameters.

### Analysis of the Questionnaire Results

**FIGURE 8:**
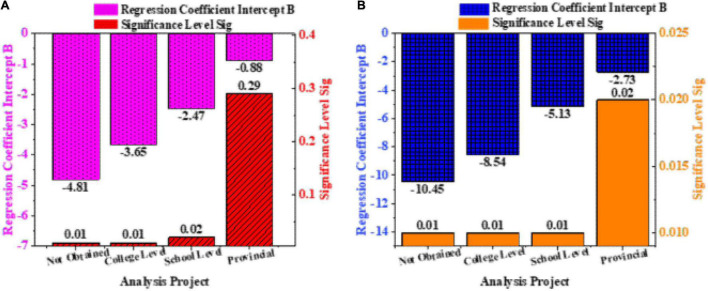
Questionnaire analysis results: **(A)** is the impact of artistic entrepreneurial performance under the support of the team and **(B)** is the impact of effective member innovation on the performance of artistic entrepreneurship.

The results of the questionnaire are counted and analyzed. By the regression analysis of the logistic model, the influencing factors of the entrepreneurial propensity of the art entrepreneurial students are discussed in the form of an entrepreneurial team. The analysis results are shown in Figure 8.

In Figure 8A, the reward results obtained by the entrepreneurial team are used as the basis. The intercept B of the regression coefficient of the entrepreneurial team that has not been rewarded is −4.81, and the significance level is 0.01. The intercept B of the regression coefficient for the hospital-level award is −3.65, and the significance level sig is 0.01. The intercept B of the regression coefficient for the school-level award is −2.45, and the significance level sig is 0.02. The intercept B of the regression coefficient for obtaining provincial awards is −0.88, and the significance level is 0.29. The intercepts of regression coefficients are all negatively affected. The level of winning is used as the dependent variable. Because of the different factors that affect performance, changes in performance cannot be fed back in real time by the scale. The intercept is calculated. With the support of the team, the higher the average score of the scale, the higher the reward level obtained. Team entrepreneurship can have clear goals, and internal individuals can better complete tasks. For provincial rewards, the sig = 0.29 > 0.05, indicating that there is no significant effect. In linear regression, the regression coefficient significance test is equivalent to the regression equation significance test. But in multiple linear regression, this equivalence does not hold. The *t*-test is to test whether each regression coefficient in the regression model is significant. Only those factors that significantly impact the dependent variable are retained in the model.

In Figure 8B, the rewards obtained by the entrepreneurial team are used as the basis. The intercept B of the regression coefficient of the entrepreneurial team that has not been rewarded is −10.45, and the significance level sig is 0.01. The intercept B of the regression coefficient for the hospital-level award is −8.54, and the significance level is 0.01. The intercept B of the regression coefficient for the school-level award is −5.13, and the significance level is 0.01. The intercept B of the regression coefficient for obtaining provincial awards is −2.73, and the significance level sig is 0.02. These four coefficients are all negative effects. The higher the average score of the scale, the higher the reward level. The reason for this phenomenon lies in the fact that in the later team entrepreneurship display, each team will conduct a concentrated competition and select high-performance teams to enter the semi-finals. In the competition, teams with high timeliness and psychological endurance can get better results. Identifying information needs is the responsibility of managers. Managers should put forward information needs according to the needs of decision-making and process control. Regarding process control, management should identify the need to use the information to support the review of process inputs, process outputs, the rationality of resource allocation, optimization of process activities, and detection of process anomalies.

### Discussion and Additional Suggestions

Through the processing and analysis of questionnaire data, this study analyzes the current situation of college students’ innovation and entrepreneurship teams in the field of art and design and the impact of various factors on the performance of college students’ innovation and entrepreneurship teams, summarizes the development trend of college students’ innovation and entrepreneurship teams, and puts forward countermeasures and suggestions.

1. Clarify the division of entrepreneurship tasks of the team and stimulate the enthusiasm of team members. The team leader should clarify the motivation of the members to participate in the innovation and entrepreneurship team of college students and arrange the project tasks in a targeted manner by analyzing the information of the members. Finally, the completion effect of college students is evaluated, and goals are set.
2. Increase the support for team entrepreneurship and improve the practice management system. By strengthening the team foundation and resource reserve, a reasonable incentive mechanism is established, and team efficacy is increased. Members and their departments are rewarded according to their needs for meeting deadlines and exceeding tasks. Team members should realize the meaning and value of their work, further stimulate their enthusiasm for work, and enhance their confidence in completing tasks.
3. Improve the level of team leadership and adjust the team’s entrepreneurial deviation in time. The implementation of the supervision mechanism is conducive to improving the concentration of the members, reducing the ambiguity of the goals, and helping the members to solve some unexpected problems during the supervision process to ensure that there is no deviation from the goals during the project execution stage. It is urged to complete the planning goals within the set time, so that the goals of the next stage can be smoothly carried out according to the original plan.
4. Strengthen team norms and correct the entrepreneurial attitude of team members. Through timely feedback of task evaluation results, the team leader can analyze team tasks and individual situations of team members according to the actual situation of target completion, reasonably arrange team activity time, maintain team members’ enthusiasm for work in combination with the team incentive system, and guarantee the probability of high efficiency in the process of team entrepreneurship.
5. The goal of talent training should be positioned accurately. First, education should be improved. Teachers’ professional skills and intellectual literacy should be strengthened. Teachers’ cultural literacy and professional knowledge level should be improved, and professional teachers of professional planning should be trained. Second, the training and cultivation of teachers’ practical ability should be strengthened, especially, the cultivation of arts and the selection of professional teachers. Only this way can educators find the right direction and opportunities on the road of talent training, and schools can train graduates with better skills and practical abilities when faced with different employment directions and needs. Only when the talent training mode is optimized, the quality of talent training is improved, and it has professional characteristics can the expected goal be achieved.

## Conclusion

At present, the number of job seekers far exceeds the industry demand. The employment problem of college graduates is becoming more and more serious. Therefore, starting from the severe entrepreneurial environment of college students, this study studies the influencing factors of entrepreneurial tendency of art graduates. Additionally, the logistic regression analysis method is used to evaluate the model of art majors in a university, and the design team entrepreneurship questionnaire is used to investigate some factors affecting team performance in the process of entrepreneurship. The results show that the logistic model can well reflect the real situation of the respondents, and the results of the questionnaire are further analyzed. In team entrepreneurship, clear goals, reasonable division of labor, high psychological endurance, and pressure resistance of each member are indispensable factors for successful entrepreneurship. This provides a certain reference basis for undergraduates in art and other majors to start their own businesses. Due to the difference in the level and type of the team’s awards, the scale cannot reflect the team’s performance in real time. This problem will be resolved in the follow-up work.

## Data Availability Statement

The raw data supporting the conclusions of this article will be made available by the authors, without undue reservation.

## Ethics Statement

The studies involving human participants were reviewed and approved by the Ningbo University Ethics Committee. The patients/participants provided their written informed consent to participate in this study. Written informed consent was obtained from the individual(s) for the publication of any potentially identifiable images or data included in this article.

## Author Contributions

All authors listed have made a substantial, direct, and intellectual contribution to the work, and approved it for publication.

## Conflict of Interest

The authors declare that the research was conducted in the absence of any commercial or financial relationships that could be construed as a potential conflict of interest.

## Publisher’s Note

All claims expressed in this article are solely those of the authors and do not necessarily represent those of their affiliated organizations, or those of the publisher, the editors and the reviewers. Any product that may be evaluated in this article, or claim that may be made by its manufacturer, is not guaranteed or endorsed by the publisher.
