# Supplementary material for: The Influencing Factors of Art Graduates’ Entrepreneurship by Logistic Regression Analysis From the Perspective of Entrepreneurial Mentality
Source: Front Psychol. 2022 Jun 22;13:870448. doi: 10.3389/fpsyg.2022.870448 (PMC9258622; doi:10.3389/fpsyg.2022.870448)
Supplement: Supplementary file 2 [file Data_Sheet_1.docx]

**Appendix**

**Questionnaire on influencing factors of the entrepreneurial intentions of art entrepreneurial teams**

Dear Sir/Madam:

Thank you for taking the time out of your busy schedule to fill out this questionnaire. This questionnaire is only to investigate the influencing factors of entrepreneurial intentions in the art entrepreneurial team, and will not be used for other purposes, nor will any personal information be disclosed. Please feel free to fill in, thank you for your cooperation!

| Team member's name:  Team name and entrepreneurial project: | | Team formation time (starting business time): |
| --- | --- | --- |
| Question No. | Content of survey question | Selection criteria:  A (very agree)  B (comparatively agree)  C (not sure)  D (some discrepancies)  E (very disapproval) |
| 1 | The team has a clear goal when starting a business. | A B C D E |
| 2 | The team has a clear mission when starting a business. | A B C D E |
| 3 | There are clear requirements for the completion of tasks of team members. | A B C D E |
| 4 | There are requirements for time configuration and implementation within the team. | A B C D E |
| 5 | Do the team's tasks ensure that individual members collaborate with each other? | A B C D E |
| 6 | Does the team have access to the necessary technical resources? | A B C D E |
| 7 | The team has an effective way to get help from outsiders. | A B C D E |
| 8 | There are regular discussions within the team on the completion of tasks. | A B C D E |
| 9 | The team regularly conducts relevant training sessions. | A B C D E |
| 10 | The team leader can have strong management skills. | A B C D E |
| 11 | Teams blame someone for internal conflict. | A B C D E |
| 12 | Every member of the team is able to work hard. | A B C D E |
| 13 | Team members will not prejudice members based on the opinions of others. | A B C D E |
| 14 | Individual members of the team have different levels of expertise or backgrounds. | A B C D E |
| 15 | A display platform where every member can acquire talents. | A B C D E |
| 16 | Teams use unnecessary cooperation to force members to collaborate. | A B C D E |
| 17 | In the event of disagreement, each member is allowed to express his or her own opinion. | A B C D E |
| 18 | Every member is able to work in the common interests of the team. | A B C D E |
| 19 | Insights are shared enthusiastically and willingly among team members. | A B C D E |
| 20 | Team leaders can lead by example. | A B C D E |
| 21 | The leader will set tasks for each member. | A B C D E |
| 22 | The leader is able to live up to his words, deeds and commitments. | A B C D E |
| 23 | The captain is able to express a positive attitude towards the members. | A B C D E |
| 24 | The team leader can guide everyone to accept common suggestions when they disagree. | A B C D E |
| 25 | Does the leader often articulate the goals and meaning of the team? | A B C D E |
| 26 | Does the leader only paint unrealistic future outcomes to members? | A B C D E |
| 27 | The team leader can sum up the experience in a timely manner when mistakes are made. | A B C D E |
| 28 | Whether the leader has a friendly relationship with each member? | A B C D E |
| 29 | Has the team leader publicly praised members who achieved results? | A B C D E |
| 30 | Team leaders appreciate different members in different ways. | A B C D E |
| Additional comments: | | |
